# Proposed Conceptual and Experience-Based Framework for Reaching an Optimal Vaccine Launch Strategy

**DOI:** 10.3390/vaccines14060535

**Published:** 2026-06-16

**Authors:** Baudouin Standaert, Marc Raes

**Affiliations:** 1 Department of Care and Ethics, Faculty of Medicine & Life Sciences, University of Hasselt, B-3590 Diepenbeek, Belgium; 2 Department of Pediatrics, Jessa Hospital, B-3500 Hasselt, Belgium; fa303885@skynet.be; 3 Department of Immunology & Infection, Faculty of Medicine & Life Sciences, University of Hasselt, B-3590 Diepenbeek, Belgium

**Keywords:** vaccine launching processes, strategies of implementation, full-value asset, economic evaluations, stakeholder selection

## Abstract

Obtaining market approval and reimbursement are necessary but not sufficient conditions for the implementation of new vaccines in high-income countries to maximize their long-term preventative value. Comprehensive pre-launch and launch-phase economic evaluations of the disease and the vaccine are necessary to support long-term public health improvement by the vaccination program. This review highlights the construction of these evaluations conceived as a plan, methods, and a tool. They can be generated by different stakeholders (e.g., payers, producers, target groups) interested in the value success of vaccination. A Vaccine Launching Value Project (VLVP) has been developed based on the experience gained from helping to launch 10 new vaccines worldwide over 15 years (2005–2020). It comprises information on the following: (1) identification of new vaccines that should require a VLVP approach; (2) country-specific characteristics of healthcare; (3) methods to assess economic values for specific stakeholders; (4) identification of the money flow in managing the disease and infection spread; and (5) optimal implementation strategies at the initiation of new vaccination programs. The benefits of applying the VLVP are illustrated using rotavirus vaccination as an example. The VLVP program starts with the development of a Broad Country Linked Inventory (Brocoli) Plan that interconnects eight baskets of information specifying a framework of activities. This is followed by the Cauliflower and Artichoke Methods to assess the vaccine value for additional key stakeholders (e.g., employers, hospital managers, working mothers, the Ministry of Finance) and the money flow amongst the payers (who pays what to whom, when, for what, and how). The evaluation process finishes with the Total Management Tool (Tomato) to identify the optimal implementation conditions at the start of a new vaccination program necessary to obtain the best long-term value for the stakeholders selected. The critical interconnections between these information blocks are discussed. This improves the positioning of a new vaccine by articulating its total economic value within a societal and public health environment over time, outside the conventional Health Technology Assessment box. The Tomato Tool emerges as the most pivotal component of the VLVP. It provides the best assurance of long-term economic value with strong sustainability support.

## 1. Introduction

Launching a new vaccine in a high-income country (HIC) in Europe typically begins after clinical trials demonstrate safety and sufficient efficacy to obtain market authorization from the European Medicines Agency (EMA) [1,2]. This is followed by country-specific reimbursement procedures, informed by the Joint Clinical Assessment (JCA) dossier, which aims to harmonize the clinical evidence across Europe [3,4,5]. Reimbursement decisions are embedded within national Health Technology Assessment (HTA) processes, where economic evaluations must demonstrate that incremental health gains justify additional costs within accepted cost-effectiveness thresholds [6,7]. These evaluations mainly rely on the clinical trial data but, particularly for vaccines, they are often extended with long-term modelling that captures delayed health benefits such as mortality reductions. The latter may substantially influence the perceived economic value [8,9].

Once a local reimbursement is granted, vaccines can enter the local market quite rapidly. However, vaccines often face a challenging launch phase as they do not address immediate patient demand and existing symptoms but aim to prevent future disease [10]. Successful vaccination depends not only on product efficacy but also on achieving high coverage rates, particularly during the early phase of implementing a new vaccination campaign to limit infection spread [11,12,13,14,15]. Early post-launch outcomes may appear satisfactory, yet suboptimal implementation at the start of a program may compromise its long-term effectiveness and weaken later assessments of the vaccine’s impact and value [16].

We argue that long-term vaccination success, once regulatory approval and reimbursement are obtained, depends on the quality of the launch strategy at the start. A poorly designed launch may undermine sustained program effectiveness, which only becomes evident through the long-term monitoring of the vaccine effect. The full expected vaccination value should not be assessed solely through the traditional clinical and pricing endpoints. Instead, the full value should be embedded into a broader framework that identifies where, for whom, and under which conditions the vaccine benefits are maximized [2,17,18,19].

Based on the experience of supporting the global introduction of different new vaccines during the past decades, we have developed a full economic, value-based, vaccine launch framework tailored for HIC settings. While numerous international and national organizations provide guidance on vaccine introduction, those approaches primarily reflect payer and recipient perspectives in countries most in need of immune prevention [20,21,22]. Our program has a focus on HICs and explicitly includes the perspective of the producer as well. This recognizes that long-term success in vaccination through sustained prevention is driven by high vaccine coverage and therefore high volume, a necessary condition for profit. It is important to note that not all vaccines require an extensive launch support program. We therefore introduce the Vaccine Launching Value Project (VLVP) as a structured and selective framework combining strategic planning, economic evaluation methods, and targeted launch execution. Applied when weaknesses in disease assessment or vaccine positioning are identified, the VLVP goes beyond the regulatory and reimbursement dossiers of the country-specific HTA process by demonstrating the full societal and economic potential of a vaccine when optimally positioned at its launch, thereby reaching long-term vaccination success. The VLVP should therefore not be considered as a replacement for the HTA but rather as an additional process allowing for new relevant value measurements outside the conventional environment, thus helping to support better societal positioning of the new intervention. To facilitate memorization of the different instruments developed, we have used common vegetable names to summarize the Plan (Brocoli), the Methods (Cauliflower and Artichoke), and the Tool (Tomato), as explained later in the text. In the next three Sections, we show the overall concept of our approach, describe concrete design and examples, and finish with a discussion of the strengths and weaknesses/limitations of the proposal.

## 2. Concept of the VLVP

History: At the beginning of the new century, three new vaccines were launched in HIC countries, the pneumococcal vaccine, the rotavirus vaccine, and the vaccine against human papilloma virus (HPV). Their economic values were assessed in many countries in Europe using a cost-effectiveness analysis (CEA) with the primary clinical endpoint of survival benefit due to mortality reduction, often expressed in quality-adjusted life-year (QALY) gain. However, due to budget constraints and the large initial investment required, many countries had difficulty implementing the three vaccines at the same time without enough certainty of the long-term benefit of each vaccine. As a result, the rotavirus vaccine was often considered a lower priority for implementation compared with the two other vaccines. This was the incentive for producers to look beyond the classical vaccine effect of reducing specific mortality caused by the infection, in an attempt to better position the vaccine in this competitive environment. For instance, the clinical trials reported a substantial reduction in hospitalizations after the introduction of the rotavirus vaccine, an important cost-saving effect [23]. That gain was poorly used at the launch time as critical value of all the benefit the vaccine could generate.

**Figure 1:**
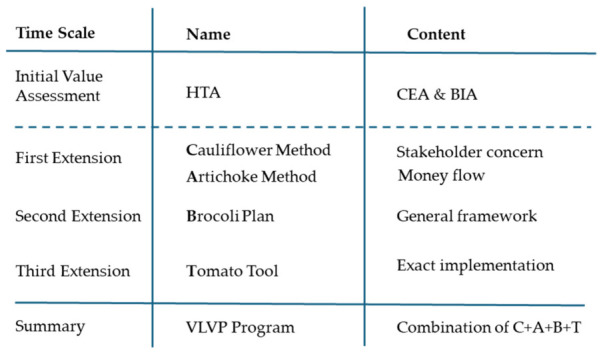
Development of the Vaccine Launching Value Project VLVP framework. HTA: Health Technology Assessment; CEA: cost-effectiveness analysis; BIA: budget impact analysis; VLVP: Vaccine Launching Value Project.

Evolution: When starting to look at ways to better position new vaccines outside the conventional measurement approaches, there were no existing plans or programs that defined methods to identify broader vaccine benefits. Our initial focus (first extension) considered two areas (Figure 1). The first aimed to identify the stakeholders potentially interested in the vaccine and to describe the specific gain offered by the vaccine to each stakeholder. In the 10 to 15 years after the launch of the three new vaccines, many different value measurement studies were instigated to explore additional vaccine benefits, including monitoring of the vaccine effect beyond the 4–5 years of conventional follow-up. Most of the studies were on the rotavirus vaccine because its uptake was limited by being a low priority in HICs. The 6-P framework of key stakeholders (population, payer, producer, provider, prescriber, politician) was described, together with the concept of the Cauliflower Method, to specify the values relevant to each stakeholder [24]. The second area aimed to move beyond the CEA as the sole method for the economic valuation of a new vaccine in a competitive public health environment. New economic assessments were developed, recognizing that healthcare was most often funded from fixed annual healthcare budgets, which is a difficult situation when the CEA results indicate that increased health benefits depend on increased expenditures. Moreover, other financial players such as third party payers (insurance) or Ministries of Finance (MOFs) used different methods to evaluate the economic value of new interventions that were not used or unknown in the healthcare environment [25]. We therefore developed the Artichoke Method to specify the existing methods of economic and financial evaluations, in addition to the conventional CEA, that could be relevant for different payers. This aimed to help build a constructive dialogue between the source of financing, typically a MOF, and the department organizing healthcare spending, typically a Ministry of Health (MOH).

Consolidation: The aim at the next stage (second and third extensions, Figure 1) was to integrate the different approaches into one overall economic value program, which we have called the Vaccine Launching Value Project (VLVP). This should include the different stakeholders’ views, different economic value assessment techniques, and methods for the correct assessment of the long-term vaccine effect to be expected based on the details of the introduction of the vaccine. The project is based on the experience of launching many new vaccines globally during the past few decades in diverse epidemiological, economic, and policy environments. Flexible thinking was required to develop context-specific economic value assessments, tailored to national decision-making processes and stakeholder priorities. This involved iterative local discussions to identify which elements of the value offered by the vaccine were essential, optional, or unnecessary, depending on the perspectives of key decision-makers and the evolving positioning of each vaccine within its market. This accumulated experience has now been consolidated into an integrated framework that underpins the VLVP, expressed as a Plan, Methods, and a Tool. These were absent when starting to develop extended value assessments of new vaccines, some 20 years ago. At that time, the vision in many countries of the role and benefit of a vaccine was limited to the idea that a vaccine must primarily reduce infant/child mortality due to the infectious disease. If mortality associated with the vaccine-targeted disease was low, there was no argument to support vaccination. These preconceived perceptions and conventions made it difficult to convince some decision-makers that vaccine benefits could extend beyond mortality reduction [26]. The initial attempts to go beyond the conventional assessments could be opportunistic, undeliberated and uncoordinated, and were therefore not always suitable or effectively communicated.

The VLVP presented here is intended as a comprehensive economic full-value assessment approach for vaccines whose initial positioning does not allow for a straightforward implementation. When required, the development of a full VLVP should ideally begin during early clinical development (Phase I–II), as initiation during the market authorization phase is often too late to influence the long-term launch success.

Construction: Five core elements define the VLVP framework. First, a new vaccine should be categorized into one of three predefined groups to assess whether a full VLVP is warranted. Group 1 (full VLVP) covers new vaccines that target endemic diseases with a well-characterized disease burden described in the epidemiological data (prevalence, incidence, at-risk populations) and established disease management pathways. Group 2 includes new vaccines developed for new emerging epidemic diseases, and Group 3 comprises new vaccines for new antigenic combinations or additional serotypes. Groups 2 and 3 often have a limited or evolving evidence base and may therefore require only a limited VLVP. This decision to develop a full VLVP also depends on disease awareness and perception among the population, healthcare professionals, public health authorities, and experts within a country, as well as on the clarity of vaccine efficacy, safety, and definition of the target population [27].

Second, a structured action plan should be developed, covering disease burden assessment, hypothesized economic values relative to current practice, supporting evidence generation (studies and publications), timelines, budgets, and prioritization. This planning approach is formalized in the Broad Country Linked Inventory (Brocoli) Plan, which provides a transparent and scalable structure for execution [28,29].

Third, optimal vaccine positioning is defined through stakeholder-specific value identification. The Cauliflower Method is a systematic approach to identify the relevant stakeholders, to tailor key messages to each group, to define the appropriate assessment methods, and to determine effective communication strategies. The “florets” of the cauliflower represent the distinct value propositions supported by the targeted evidence. For instance, demonstrating reduced work absenteeism among parents following vaccination against pediatric diarrheal disease could also be beneficial information for employers and social security organizations. Practical examples illustrate how stakeholder-specific benefits can be selected and substantiated [24,30].

Fourth, the Artichoke Method considers the different payers in the healthcare system using different evaluation references focused on money only and on the money flow. It searches for alternative methods of evaluation, and we selected the artichoke as a name because of its composition of bract leaves; this differs from the florets in the cauliflower where money is only one element. The Artichoke Method considers the money flows involved in managing the disease and the spread of infection (i.e., identifying who pays what, when, where, for what, and to whom). It includes techniques such as budget optimization, fiscal health modelling, and the use of social accounting matrices [31,32].

Fifth, and most importantly, the VLVP incorporates an explicit vaccine implementation strategy, intended to ensure that the vaccination program achieves and maintains its intended preventative effects over time. This Total Management Tool (Tomato) defines the optimal conditions for vaccine deployment at the start of a new vaccination campaign. It identifies the key interactions between the disease characteristics and the vaccine properties that determine the short- to long-term population-level impacts [33]. Without such an implementation strategy, the value propositions defined in the earlier steps cannot be reliably demonstrated to the different stakeholders who may be interested in the value assessment of the new vaccine. For instance, if a new vaccine does not have an optimal launch strategy to ensure a high initial coverage, its effect on reducing disease incidence (via direct and indirect effects) may be smaller than could be achieved with an optimal strategy, which in turn may mean that the number of parents having to take time off work to care for a sick child remains higher than could be achieved with an optimal strategy, with consequently less reason for an employer to pay for vaccination.

## 3. Design with Examples

### 3.1. Assessment of Need for VLVP

The first step in applying the VLVP is to determine whether full VLVP development is essential for a new vaccine. Developing a VLVP is a resource-intensive, multi-year effort requiring dedicated budgets, milestones, and pre- and post-launch activities. To ensure that these efforts are focused where most needed, new vaccines coming onto the market should be categorized based on disease type (endemic [Group 1] vs. epidemic [Group 2]) and vaccine formulation characteristics (e.g., new combinations or updated serotypes, Group 3), which determine the level of VLVP support needed (Table 1).

Based on our experience, we recommend that only those vaccines targeting endemic diseases with established epidemiology and disease management pathways (Group 1) are typically candidates for a full VLVP (Table 1). Vaccines developed for an epidemic response (e.g., COVID-19) (Group 2) and new combinations or serotype updates (Group 3) often rely on urgency or pre-existing evidence and therefore cannot justify the effort of a full VLVP development. For these products, a limited VLVP approach may be sufficient.

Among the list of endemic-disease vaccines mentioned in Table 1, the rotavirus vaccine has required the most comprehensive application of a VLVP. The rotavirus vaccine was not perceived as a priority in HICs, given the low mortality rate, competition between vaccines, defining vaccination priorities at launch, and uncertainty regarding the economic returns [34]. The other endemic-disease vaccines in Table 1 only applied selected VLVP components, due to the strong clinical evidence of the vaccine effect in the trial results, the limited competition, or the data constraints that reduced the development of a full VLVP framework.

### 3.2. Brocoli Plan

Advances in health economic evaluation methods, spanning value, budget, and fiscal perspectives, have expanded the toolkit available for the economic value assessment of vaccines [31,35]. However, the systematic application of these methods in real-world decision-making is challenging. As vaccine price negotiations are ultimately anchored by the value assessed, a more structured approach is required to guide evidence generation.

**Figure 2:**
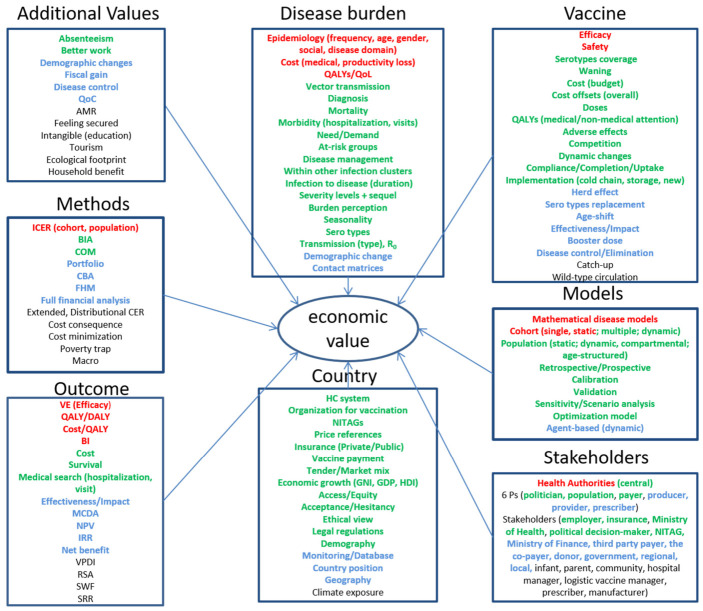
Brocoli Plan illustrating elements that can influence the economic value of a new vaccine (red: fundamental; green: essential; blue: critical; black: interesting). AMR: anti-microbial resistance; BI: budget impact; BIA: budget impact analysis; CBA: cost–benefit analysis; CER: cost-effectiveness result; COM: constrained optimization model; DALY: Disability Adjusted Life Year; FHM: fiscal health modelling; GDP: Gross Domestic Product; GNI: Gross National Income; HC: healthcare; HDI: Human Development Index; ICER: incremental cost-effectiveness result; IRR: internal rate of return; MCDA: multi-criteria decision analysis; NITAG; National Immunization Technical Advisory Group; NPV: net present value; QALY: quality-adjusted life year; QoC: quality of care; QoL: quality of life; R_o_: basic reproduction number; RSA: Risk Sharing Agreement; SRR: Social Rate of Return; SWF: Social Welfare Function; VE: Vaccine Efficacy; VPDI: Vaccine-Preventable Disease Incidence.

The Broad Country Linked Inventory (Brocoli) Plan provides such a structure. It represents a comprehensive economic evaluation blueprint encompassing all the potential value drivers for a vaccine (Figure 2). While rarely fully developed due to the constraints of time, cost, and feasibility, the Brocoli Plan maps the full-value landscape and helps to prioritize the generation of evidence. It includes all the elements necessary to construct and apply the Cauliflower and the Artichoke Methods. However, it does not focus on the Tomato.

Importantly, additional evidence of value is not automatically translated into a higher reimbursement price. The objective is rather to inform decision-making in other contexts or countries to position the vaccine better as a valuable intervention. This can be illustrated by the study on the improvement of quality-of-care in Belgium for rotavirus vaccination that later proved to be critical information for France. In Belgium, the study results did not result in any increase in vaccine price or sales.

Figure 2 organizes the vaccine value assessment into eight interconnected domains of disease burden, vaccine characteristics, modelling approaches, stakeholders, country context, additional values, analytical methods, and outcome measures. The items presented in each domain in Figure 2 are shown in different colors to indicate their importance (red is fundamental information, green is essential, blue is critical, black is interesting). Many links across the domains are not shown due to space limitations. For instance, the disease burden data are needed as input information into the model construction, which in turn is critical for the method analysis. Making a comprehensive economic value evaluation of a new vaccine to understand when, how and for whom its potential impacts apply could therefore be complex and cumbersome. In vaccine implementation, much is dependent on the characteristics of the country and its healthcare organization. If all the red and green information in each domain for a country under study is known and mapped, it should allow an economic value assessment of the vaccine to be made with sufficient confidence to inform the definition of the reimbursement price. Consequently, the first focus of the economic analysis method is on the incremental cost-effectiveness ratio (ICER) with a budget impact analysis (BIA) and constrained optimization models (COMs). A cost–benefit analysis (CBA) and fiscal health modelling (FHM) are positioned as second-order investigations [36].

Clinical trials remain the starting point for the value assessment, focusing primarily on safety and efficacy under controlled conditions. Broader epidemiological, societal, and country-specific data are rarely collected within these first trials and must be generated separately. Additional outcomes—such as patient-reported outcomes (PROs) or disease-specific complications—are incorporated only when differentiation or market access requires them, as demonstrated in the zoster vaccine development program [37].

The failure to identify relevant additional value domains early in the development may compromise the launch success. For rotavirus vaccination, insufficient attention was initially paid to the effects on hospital capacity and on work absenteeism among parents, factors that proved to be highly relevant to hospital managers and employers, respectively [23,38,39,40]. Earlier recognition of these benefits would probably have favored alternative economic analytical approaches, such as CBA. However, when the rotavirus vaccine was launched in the HIC, there was no Brocoli Plan available that could have helped in identifying these critical points. A staged approach to value assessment is therefore recommended: first, optimize the clinical trial data collection for launch-critical outcomes; second, identify and generate additional value evidence relevant to subsequent markets [41]. A well-developed Brocoli Plan enables the systematic identification of where and how additional vaccine benefits may materialize.

### 3.3. Cauliflower Method

Once the Brocoli Plan is defined, the Cauliflower Method can be applied to translate the vaccine value into stakeholder-specific propositions. Each “floret” represents a stakeholder group, its relevant value perspective, and the evidence required for its support. Using rotavirus vaccination as an example, up to 17 stakeholder groups were identified as potential beneficiaries (Table 2). Unlike therapeutic interventions, vaccines generate value through prevention, often in the absence of explicit clinical/symptom demand [24,36,42]. This creates an additional requirement for education and framing of the benefits. Stakeholders range from patients and healthcare providers to employers, payers, and Ministries of Finance (grouped into six types, the 6 Ps).

**Figure 3:**
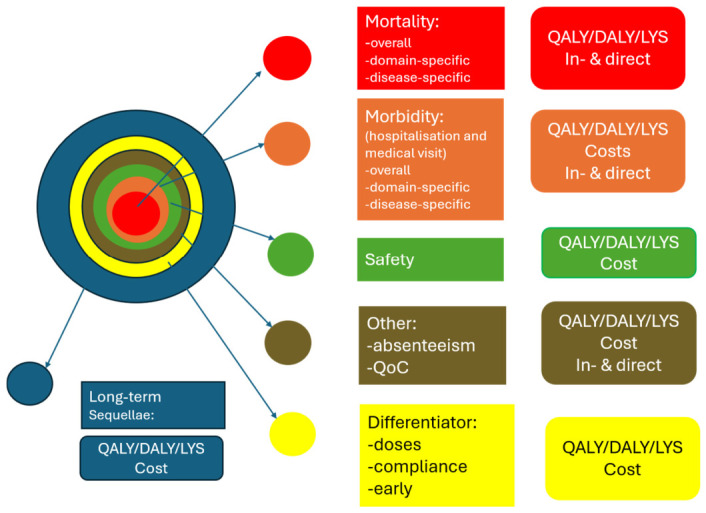
Defining the different potential value measures for the rotavirus vaccine. QALY: quality-adjusted life year; DALY: Disability Adjusted Life Year; QoC: quality of care; LYS: Life Year Saved.

Vaccines may generate a broad spectrum of benefits—up to 24 distinct value elements—across clinical, societal, implementation, safety, and differentiation domains [43]. The benefits are enumerated and regrouped into these specific domains in Figure 3. The evaluation shifts from the individual-level value to the population-level public health benefit, marking a transition from value-based healthcare to value-based public health. Given the diversity of the benefits, prioritization and aggregation become essential, with a multi-criteria decision analysis (MCDA) offering a potential approach for summation [44,45].

For rotavirus vaccination, the Cauliflower Method supported value generation in six non-clinical domains: work absenteeism reduction for employers, quality-of-care improvements for hospital managers, time benefits for working mothers, fiscal gains for the Ministry of Finance, dosing advantages for payers, and portfolio management for public health decision-makers [32,39,46,47]. However, the value effects may also evolve dynamically: hospital capacity, freed by the introduction of earlier vaccines, has later increased the perceived value of respiratory syncytial virus (RSV) vaccination, particularly in Belgium [48].

### 3.4. Artichoke Method

In contrast to the Cauliflower Method that focuses on the value by the stakeholder type, the Artichoke Method focuses only on money and payment. As indicated in Table 2, six different payer entities were identified. They all receive or spend money to manage the disease, and the expenditure could be different before and after the introduction of the vaccine as a result of the reduced infection spread. The critical issue is to understand who gains or loses most, and over what timescale. We were involved in the application for rotavirus vaccination in the Netherlands, where there were difficulties in approving the rotavirus vaccine because the decision-makers’ initial attention was on mortality reduction [32,49]. A discussion with the Ministry of Finance regarding the use of a social accounting matrix evaluation helped to open the discussion on the total value benefit the vaccine could generate but was initially not enough to convince the Ministry of Health. However, since 2024, the rotavirus vaccine has been fully reimbursed in the Netherlands.

### 3.5. Tomato

While value assessment and stakeholder positioning are essential, vaccines can only achieve their full impact when implemented under optimal conditions. The Total Management Tool (Tomato) addresses these critical implementation dimensions.

**Figure 4:**
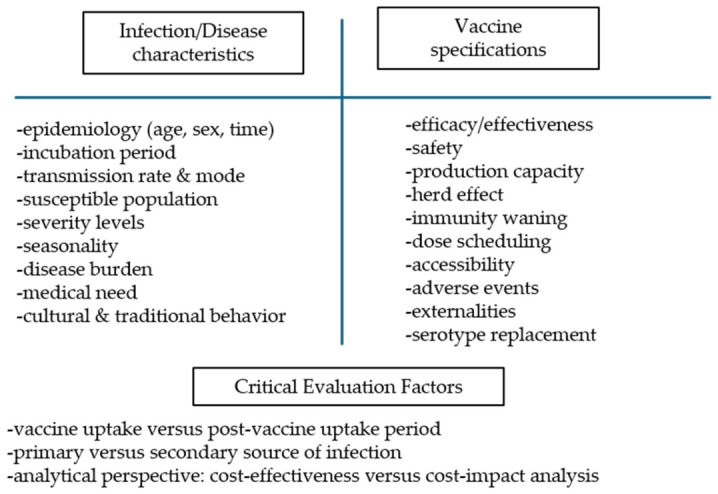
The elements defining Tomato.

Optimal vaccine performance depends on two interacting domains (Figure 4). The disease-specific factors include epidemiology (age and sex spread by time unit), incubation period, transmission dynamics (who infects whom, where, and when), susceptible populations, seasonality, disease burden and severity level, cultural and traditional behavior, and unmet medical need. The vaccine-specific factors include efficacy/effectiveness, safety, production capacity, herd effects, waning immunity, dosing schedules, accessibility, adverse events, externalities, and serotype replacement. In total, 19 interacting factors must be aligned within a narrow age and time window at the launch of a new vaccine to maximize the population-level impact in the short to long term. A failure to optimize these conditions can compromise the long-term success. Due to the seasonality of the infection in Belgium (January to end of March), the delayed introduction of rotavirus vaccination did not optimize the early herd immunity effect, allowing infection to shift to older age groups and resulting in lower long-term vaccine economic performance compared with countries that launched earlier in the season, such as Finland and the UK [50,51].

Through pre-launch scenario modelling, the Tomato allows the estimation of the health and economic outcomes under alternative implementation strategies. The key analytical considerations include differentiating uptake and post-uptake dynamics, identifying primary and secondary infection sources, and selecting appropriate analytical perspectives (cost-effectiveness vs. cost–impact analysis) that capture both the direct and indirect effects of vaccination [52].

## 4. Discussion

The VLVP framework is an integrated approach consisting of four components: the Brocoli Plan maps a comprehensive set of value-relevant elements grouped into several structural analysis domains that can be used to generate conventional and novel value characteristics; the Cauliflower Method assesses extended value propositions across multiple stakeholders; the Artichoke Method searches for alternative economic or financial evaluations; and Tomato defines a detailed vaccine implementation strategy for the initiation of a new vaccination program.

In retrospect, this VLVP approach has been applied most extensively to the rotavirus vaccination program. The VLVP development was driven by the initially low perceived burden of rotavirus disease in HICs and the limited enthusiasm indicated by a low vaccine uptake. This situation stimulated the introduction of portfolio management for local immunization policies, whereby prioritization among competing vaccines is guided by single or composite decision criteria under the constraints of budget, logistics, and supply [47,53]. A recent assessment of the benefit of this more extended approach received positive comment [54].

Traditional infectious disease modelling has largely focused on predicting transmission dynamics with and without the control intervention. It may often lack a sufficient exploration of the underlying drivers of disease impact, particularly when the vaccines are introduced under new or poorly understood conditions. This limitation was evident during the COVID-19 pandemic, when early models were characterized by high uncertainty in estimating both direct and indirect vaccine effects. When the COVID-19 vaccines became available, the main initial focus of the vaccination program was to reduce COVID-19-specific mortality and remove the need for non-pharmaceutical interventions such as periodic lockdowns and restrictions on activities. There was never an intention to aim for the maximum control of infection spread, potentially leading to infection elimination. This may have contributed to the development of the current situation, in which COVID-19 has reached an endemic state in many regions, with vaccines providing only partial control of infection spread [55].

To improve the value assessment of new vaccines, we argue for a more systematic, data-driven evaluation at the outset of new vaccination programs, particularly for endemic infections and diseases. Such evaluations should explicitly define what constitutes success; for whom; how and by when it can be reached and measured; and the conditions under which it may fail in the short or long term. For instance, vaccine launch strategies based solely on price–volume agreements or gradual coverage expansion frequently fall short in delivering sustained health benefits. In contrast, well-resourced campaigns aimed at a rapid achievement of high vaccine coverage have consistently produced stronger public health and economic returns even over the long term, such as rotavirus vaccination in Finland and UK. Ironically, the long-term evaluation of a vaccine effect is often missing for countries that have implemented a highly successful vaccination program, because the disease levels fall so low that there is little to measure.

Reflecting on the introduction of rotavirus vaccination in Europe two decades ago, it is evident that the analytical and modelling tools at that time were limited, compared with what is currently available [56,57,58]. Advances in infectious disease modelling and vaccine technology mean that vaccine launches can now be planned and executed more effectively, generating greater long-term impacts and return on investment estimates with more reliable precision—provided that adequate preparatory work has been undertaken prior to market introduction.

Not every vaccine requires the full deployment of all the VLVP components. Among the five tools described here —the VLVP decision instrument, the Brocoli Plan, the Cauliflower Method, the Artichoke Method, and the Tomato— the Tomato may be the most critical for long-term success. The large-scale introduction of a new vaccine into a population causes substantial perturbation in the existing host–pathogen infection equilibrium. Vaccination creates a sudden imbalance, and under selective pressure, pathogens tend to exploit pathways of least resistance and may persist silently in transmission niches until sufficient susceptible contacts accumulate to trigger renewed outbreaks [59]. The Tomato is specifically designed to prevent this process by identifying and disrupting such transmission “islands” early and effectively.

The Brocoli Plan, the Cauliflower Method and Artichoke Method primarily serve as facilitators of vaccine introduction, acceptance, endorsement, and reimbursement by addressing a broad audience across society. They aim to enrich the understanding of the full value of vaccination and support stakeholder engagement. At the same time, a well-developed Tomato—supported by extended disease and transmission modelling—can reveal hidden risks and dynamics, such as delayed herd effects or emerging secondary disease peaks, thereby informing and refining the value propositions identified in the Brocoli and Cauliflower components.

The complete VLVP process may therefore serve as a reference framework for future vaccines, particularly those targeting endemic diseases with well-characterized epidemiology and management pathways, such as RSV. In recent years, a rapid influx of new vaccines has complicated prioritization decisions for policymakers, particularly in the absence of a comprehensive picture of the total vaccine value at launch. A structured VLVP approach can help address this challenge by clarifying the value across multiple dimensions.

This leads to a broader discussion about value-based public health. Unlike traditional value-based healthcare—which focuses on intervention pricing and the treatment of existing diseases—value-based public health requires a shift in perspective, action, and time horizon [60]. The emphasis changes from reacting to disease-specific morbidity to proactively preventing future harm. The primary beneficiary of public health is not the individual patient but society as a whole, through improved population-level health. Whereas treatment strategies aim to reduce volume while increasing value, public health interventions must find an optimal balance between volume and value, prioritizing risk reduction rather than symptom alleviation. This proactive approach is inherently more complex to implement. Adopting this broader perspective reveals a multidimensional concept of value that extends beyond health gain. The vaccine value may accrue at multiple levels, including individual, household, community, healthcare system, society, payer, and government. Value-based public health therefore resembles a squid, with multiple tentacles operating simultaneously across diverse domains to maximize the overall benefit of preventive interventions [61]. Rotavirus vaccination illustrates such multiple, sequentially beneficial effects of vaccination. It immediately caused a dramatic reduction in rotavirus-related hospitalizations, which indirectly improved the quality of care in pediatric wards during winter disease peaks. This was followed by a reduction in absenteeism of working mothers, who were willing to co-pay for the vaccine, and social security organizations saw less spending on sick leave. Each of these five elements (hospitalization reduction, improvement in quality of care, reduced work absenteeism, willingness to co-pay for the vaccine by the user, gains in social security spending), was investigated separately, but were effectively linked to each other and provided benefits for many different additional stakeholders (parents and family, healthcare workers, reimbursement authorities, health insurances, employers, and social security agencies).

The VLVP approach may have some limitations. As noted, not all vaccines require such an extensive framework due to the considerations of efficiency, time, and budget. For vaccines targeting well-understood diseases with well-demonstrated effects, adherence to a robust Tomato implementation strategy may be sufficient to ensure successful and sustainable vaccination outcomes. Other limitations include data availability and accessibility for analysis, or a lack of a clear overview setting out the expected consequences of vaccination, including timescales and measurement methods. Identifying additional benefits requires creativity in understanding the processes and consequences of the activities and identifying the specific items to be measured and quantified. The Brocoli Plan helps to address this by demonstrating the consequences that may be expected across a range of domains and how they may be reported.

Finally, it should be stressed that the VLVP was not conceived to increase the reimbursement prices of new vaccines but to improve the positioning of new vaccines through a more complete value assessment at the societal level. Vaccine pricing should be linked to the budget available, the high vaccine volume rapidly reached by investment in maximizing early coverage, and a reasonable profit margin for the producers that allows for the continuation of research and development programs. It should not be based on the extended full-value assessment, as that may be too costly to maximize the chances of its long-term success [62].

## 5. Conclusions

The VLVP was developed as a comprehensive program for assessing the full economic value of a new vaccine to support market launch decisions in HIC. It is grounded in the extensive experience gained from the global introduction of new vaccines during the past decades. The approach is structured around five interdependent information units that mutually reinforce one another. Among these, the Tomato—which defines the vaccine implementation strategy at the start of a vaccination program—emerged as the most important and broadly applicable component across the vaccine launches. It offers the strongest assurance for long-term vaccination success. The other instruments are complementary and are particularly valuable when a vaccine’s value proposition is not immediately apparent or not well understood within the context of national public health policy.

## Figures and Tables

**Table 1:** The three vaccine groups that do or do not need a full VLVP.

Full VLVP	No Full VLVP		
Disease Type		Vaccine Characteristics	
Group 1	Group 2	Group 3	
New Endemic	New Epidemic	New Combination	Adjusted Serotypes
Rotavirus	COVID-19	Tetanus, Diphtheria, Pertussis	pneumococcal bacteria
Human papilloma virus			Influenza
Malaria			
Meningococcal B			
Herpes Zoster			
RSV			

RSV: respiratory syncytial virus; VLVP: Vaccine Launching Value Project.

**Table 2:** List of stakeholders to be considered in a vaccination program.

Stakeholder Type	Identification
Population	Subject/Individual (patient, carer)
	Household
	Community
	Society
	Next generation
Payer	Ministry of Health
	Ministry of Finance
	Third party payer
	Co-payment
	Employer
	Employee
Producer	Industry(s)
Prescriber	Physician (1st & 2nd line)
Provider	Pharmacist
	Wholesaler
	Product manager in EPI
Politician	Policy view

EPI: Extended Program for Immunization.

## Data Availability

Not applicable.
